# Intermittent parathyroid hormone 1–34 induces oxidation and deterioration of mineral and collagen quality in newly formed mandibular bone

**DOI:** 10.1038/s41598-019-44389-8

**Published:** 2019-05-29

**Authors:** Yohsuke Yoshioka, Eiki Yamachika, Makoto Nakanishi, Tadashi Ninomiya, Sho Akashi, Sei Kondo, Norifumi Moritani, Yasuhiro Kobayashi, Tatsuo Fujii, Seiji Iida

**Affiliations:** 1 Department of Oral and Maxillofacial Reconstructive Surgery, Okayama University Graduate School of Medicine, Dentistry and Pharmaceutical Sciences, 2-5-1 Shikata-cho, Kita-ku, Okayama, Japan; 2 Department of Oral and Maxillofacial Reconstructive Surgery, Okayama University Hospital, 2-5-1 Shikata-cho, Kita-ku, Okayama, Japan; 3 Department of Applied Chemistry, Graduate School of Natural Science and Technology, Okayama University, 1-1-1 Tsushima-Naka, Kita-ku, Okayama, Japan; 4 Department of Anatomy, Nihon University School of Dentistry, 1-8-13 Kanda-Surugadai, Chiyoda-ku, Tokyo Japan; 5 Division of Hard Tissue Research, Institute for Oral Science, Matsumoto Dental University, 1780 Gobara Hirooka, Shiojiri, Nagano Japan

**Keywords:** Implants, Structural biology

## Abstract

Intermittent parathyroid hormone (PTH) administration is known to promote bone healing after surgical procedures. However, the mechanism and influence of PTH on the mineral and collagen quality of the jaw are not well understood. Most studies have focused on analyzing the bone density and microstructure of the mandible, and have insufficiently investigated its mineral and collagen quality. Oxidative stress activates osteoclasts, produces advanced glycation end products, and worsens mineral and collagen quality. We hypothesized that PTH induces oxidation and affects the mineral and collagen quality of newly formed mandibular bone. To test this, we examined the mineral and collagen quality of newly formed mandibular bone in rats administered PTH, and analyzed serum after intermittent PTH administration to examine the degree of oxidation. PTH administration reduced mineralization and worsened mineral and collagen quality in newly formed bone. In addition, total anti-oxidant capacity in serum was significantly decreased and the oxidative-INDEX was increased among PTH-treated compared to vehicle-treated rats, indicating serum oxidation. In conclusion, intermittent administration of PTH reduced mineral and collagen quality in newly formed mandibular bone. This effect may have been induced by oxidation.

## Introduction

Bone strength is evaluated from bone density and bone quality, where bone quality is defined by its structural and material characteristics^1^. Structural characteristics are evaluated based on the macroscopic structure of bone, the microscopic structure of cancellous trabecular bone, and cortical bone porosity. In contrast, material characteristics are evaluated based on the extent of mineralization, mineral quality, and collagen quality^1,2^. While the structural characteristics of bone substantially depend on remodeling, material characteristics vary markedly according to oxidative stress, in addition to bone remodeling^3,4^. Oxidative stress stimulates the differentiation and active factors of osteoclasts, and induces the production of advanced glycation end products (AGEs), which cause a deterioration in material characteristics^3,4^.

Intermittent administration of parathyroid hormone (PTH) causes high bone turnover with superior osteogenesis, and thereby increases bone density and improves the structural characteristics of the femur and spine. Intermittent administration of PTH has therefore been adopted for the treatment of osteoporosis^5^. Many clinical and animal studies indicate that intermittent administration of PTH promotes bone healing^6–9^. In addition to improving bone density and the structural characteristics of newly formed vertebrae and long bone after injury, which are formed by endochondral ossification^6,7^, intermittent administration of PTH also improves bone density and the structural characteristics of newly formed uninjured skull and jaw bone, which is formed by intra-membranous ossification^8,9^. However, the effect of intermittent administration of PTH on the material characteristics of new bone is unclear. To our knowledge, the only study to have reported the influence of PTH on these characteristics was conducted in a tibial fracture model^10^, and showed that PTH altered the material characteristics of the newly formed tibia bone^10^. However, the effect of PTH administration on the material characteristics of newly formed jawbone, which has a different ossification mode from the tibia, is unclear. In addition, the jaw characteristically differs from other bones, exhibiting faster bone turnover and experiencing stronger forces, such as occlusal forces. Accordingly, drugs may have a different impact on the jaw compared to other bones.

Improving surgical outcomes in the maxillofacial area requires an understanding of bone quality^11–13^. This is especially true for dental implant surgery: in these procedures, surgical success is greatly dependent on bone quality, and an understanding of bone mass and bone quality is therefore indispensable^12,13^. Because the bone mass and bone quality of the jaw are deteriorated in patients with osteoporosis, dental implant treatment in these patients is limited, and the success rate of dental implant treatment is reportedly low^14,15^. Life expectancy is increasing however, and the need for dental implant surgery in these patients is expected to increase. Several studies have therefore aimed to improve depreciated bone mass and bone quality using PTH administration^16,17^. PTH increases bone mass and improves its structural characteristics, and is expected to increase the availability of dental implant treatment and enable stable osseointegration after dental implant surgery^16,17^. However, the effect of PTH administration on the material characteristics of newly formed bone in the jaw is currently unclear. Integration of a dental implant into existing bone can be achieved by intervening in bone formation and the ongoing bone healing process. Adapting PTH to implant treatment therefore requires a precise understanding of the effect of PTH on both the structural and material characteristics of newly formed jawbone.

Raman microspectroscopy is a powerful tool for analyzing the material characteristics of bone. The Raman microspectroscope is a non-contact, non-destructive, and label-free device, and the technique requires few pre-treatments of bone samples to comprehensively analyse specific molecules. Raman spectroscopy can be used to multilaterally evaluate the material characteristics of bone by measuring several parameters, including the degree of calcification, crystallinity, concentration of carbonates and phosphates, and completeness of the collagen structure^18–20^.

In this study, we analyzed the effect of PTH on the balance between oxidative stress and anti-oxidant capacity, and demonstrated its influence on the material characteristics of new mandibular bone.

## Materials and Methods

All animal studies were approved by the Institutional Animal Care and Use Committee of Okayama University, and experiments conformed to all guidelines and regulations for the protection of welfare of animals (protocol No. OKU-2018325).

### Animals

**Figure 1:**
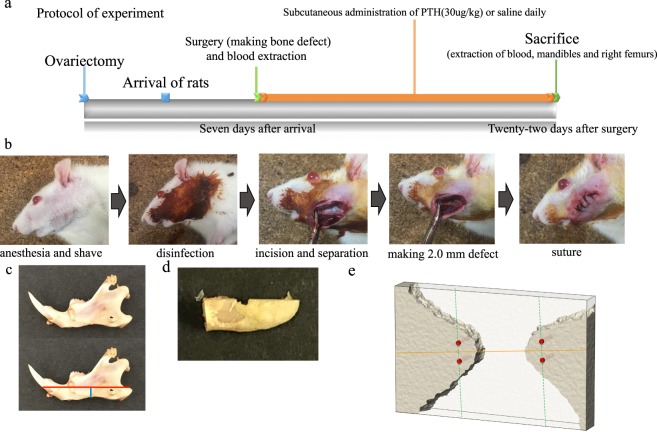
Experimental procedure. (**a**) Experimental protocol. The protocol shows the process up until the blood and mandibles were extracted. (**b**) Surgical procedure. Photographs illustrating the surgical procedures used in the mandibular defect rat model. An appropriate area on the left mandible was shaved and disinfected with 10% povidone iodine. The left side of the lateral aspect of the mandibular ramus was incised to the subperiosteal level, and subperiosteal peeling to the lower and posterior margins of the mandible was performed to clearly reveal the surgical field. A point located 4 mm from the posterior margin and 2 mm from the lower margin on the left side of the mandible was drilled bicortically using a 2-mm pin vise. The wound was closed with sutures. (**c**) Extracted mandible and cutting line for Raman analysis. First, the mandibles were cut using a diamond saw parallel to the lower mandibular plane so as to pass through the drilled hole (red line). Second, another cut was made anterior to the drilled hole (blue line). The cut mandible was polished with an Al_2_O_3_ polishing disk, including the center of the hole, to smoothen the cut surface. (**d**) Mandible after cutting and polishing for Raman analysis. Mandibles after cutting and polishing were subjected to Raman analysis. (**e**) Measurement points for Raman analysis. The outer circumference at a distance of 90 μm from the outer periphery of the drilled hole was examined using an optical electron microscope attached to the Raman microspectroscope. A straight line (yellow line) connecting the mesial and distal edges of the drilled hole was drawn across the smallest diameter, and a second line was drawn perpendicular to the first (green dotted line) from outside the 90-μm circumference to the mesial and distal edge of the drilled hole. Four points (red dots) along the perpendicular line (green dotted line) were used as measurement points.

The experimental protocol is shown in Fig. 1a. A total of 12 female Wistar ovariectomized rats aged 8 weeks were obtained from Japan SLC, Inc. (Hamamatsu, Shizuoka, Japan). Rats were ovariectomized four days before arrival in our department. The rats were maintained in a temperature- and humidity-controlled room with a 12-hour day/night cycle and fed food and water *ad libitum*.

### Dosing regimens and surgical technique

After acclimation for 7 days, they were assigned to two groups: (1) ovariectomized rats treated with intermittent parathyroid hormone 1–34 (PTH rats, n = 6); and (2) ovariectomized rats given normal saline as vehicle (OVX rats, n = 6). All rats underwent blood extraction and surgical procedures (Fig. 1b) under general anesthesia (combination of medetomidine, midazolam and butorphanol mixture)^21^. First, 2 ml of blood was collected from the lateral tail vein of all rats. The rats were then shaved and disinfected with 10% povidone iodine. The left surface of the lateral aspect of the mandibular ramus was sectioned to the subperiosteal level, and subperiosteal peeling to the lower and posterior margins of the mandible was conducted to clearly show the surgical field. At 4 mm from the posterior margin and 2 mm from the lower margin on the left aspect of the mandible, a hole was drilled bicortically using a 2-mm diameter pin vise. The wound was sutured closed. From one day after the surgical procedure, PTH rats were subcutaneously administered PTH (1–34) at 30 μg/kg body weight daily, while OVX rats were subcutaneously administered the same volume of saline. Twenty-two days after the surgery, all rats were sacrificed under general anesthesia (medetomidine, midazolam and butorphanol mixture)^21^, and 2 ml of blood was collected from the lateral tail vein for analysis of oxidation. Subsequently, the mandible and right femur were removed, then stored in a 70% ethanol solution at 4 °C in preparation for micro-computed tomography (microCT), peripheral quantitative computed tomography (pQCT), and Raman analysis.

### Analysis of the right femur using microCT

Undecalcified right (Rt.) femur bones (OVX rats, n = 6; PTH rats, n = 6) were subjected to three-dimensional microCT (Scan Xmate-A080, Comscan Tecno Co. Ltd. Yokohama, Japan) analysis. After the CT images were constructed into three-dimensional images, bone morphometric analysis was conducted with analysis software (TRI/3D-BON, Ratoc System Engineering Co. Ltd. Tokyo, Japan). We selected trabecular bone in the distal metaphysis of the femur as sampling site, located 1.5 mm to 3.5 mm from the growth plate. We measured the morphometric parameters of bone volume (BV/TV; %), trabecular thickness (Tb. Th; μm), trabecular number (Tb. N; 1/mm), and trabecular separation (Tb. Sp; μm).

### Analysis of the right femur using pQCT

Undecalcified Rt. femur bones (OVX rats, n = 6; PTH rats, n = 6) were evaluated using pQCT, and bone mineral density (BMD; mg/cm^3^) was measured. Four regions, regions 1, 2, 3 and 4, were defined as points at 1, 2, 3 and 12 mm from the growth plate to the diaphysis, respectively. The BMD at these regions was measured using pQCT (Norland/Stratec XCT Research SA+; Stratec Medizintechnic GmbH, Pforzheim, Germany).

### Analysis of the mandible by microCT

Undecalcified mandibles (OVX rats, n = 6; PTH rats, n = 6) were subjected to three-dimensional microCT analysis. After these CT images were constructed into three-dimensional images, bone morphometric analysis was conducted using TRI/3D-BON. An 800 μm × 800 μm square from the center of the drilled area on the mandible was chosen as sampling site. The same morphometric parameters as those for right femur analysis were measured. In addition, images of sagittal sections of the mandible including the drilled hole were extracted, and the area of the hole (μm^2^) was measured using analysis software (Axio Vision 3.1, Carl Zeiss Co. Ltd, Munich, Germany).

### Analysis of the mandible using pQCT

A rectangular region with long edges at a distance of 1.3 mm from the center of the drilled hole in the mandible (OVX rats, n = 6; PTH rats, n = 6) was scanned using pQCT, and BMD (mg/cm^3^) was measured.

### Mapped image of bone quality of the mandible using Raman microspectroscopy

Undecalcified mandibles (OVX rats, n = 6; PTH rats, n = 6) were inspected by Raman microspectroscopy (NRS-5100; Jasco Co. Ltd, Tokyo, Japan) after analysis using microCT and pQCT. Before measurement, the mandibles were processed in several steps. First, the mandibles were cut using a diamond saw (BS-300C, Meiwafosis Co. Ltd, Tokyo, Japan) parallel to the lower mandibular plane so as to pass through the drilled hole. Second, another cut was made anterior to the drilled hole (Fig. 1c). Third, the cut mandible was polished with an Al_2_O_3_ polishing disk (MA-200D, Misashino Denshi Co. Ltd, Tokyo, Japan), including the center of the hole, to smoothen the cut surface (Fig. 1d). These processed mandibles were subjected to Raman microspectroscopy. The Ar-ion laser power (wavelength: 532 nm) for these measurements was 100 mW. Raman spectra were measured twice with an acquisition time of 60 s each, and accumulated. Analysis was limited to the Raman scattering region between 800 and 1800 cm^−1^. Quantitative maps of the distribution of newly formed bone (300 μm × 120 μm) within the sample area, including the drilled hole, were obtained using a scanning step size of 10 μm. Further, the outer circumference at a distance of 2 mm and 90 μm from the outer periphery of the drilled hole was investigated using an optical electron microscope which was attached to the Raman microspectroscope. A straight line connecting the mesial and distal edges of the drilled hole was drawn through the smallest diameter of the hole (Fig. 1e), and another line perpendicular to the first was drawn from outside the 90-μm circumference to the mesial and distal bone edges using the Raman microspectroscope software (Fig. 1e). Four points on the perpendicular line were used as measurement points of 90 μm (Fig. 1e). Subsequently, four spectra per specimen were randomly obtained at w2 mm and 90 μm from the bone edge around drilled hole. Peaks were assigned and interpreted based on previously described methods^18–20^. The following commonly used Raman bone metrics were calculated at each measurement point:

- Mineral/matrix ratio = intensity ratio between v_1_PO_4_ (930–980 cm^−1^) and amide I (1620–1700 cm^−1^). High values indicate more mineralization.
- Crystallinity = inverse of the full-width at half-height (FWHH) of the v_1_PO_4_ band.
- B-type carbonate substitution = intensity ratio between B-type CO_3_^2−^ (1050–1115 cm^−1^) and v _1_PO_4_ bands. High values indicate more B-type carbonate substitution.
- Monohydrogen phosphate content = intensity ratio between the v_3_HPO_4_^2−^(1003 cm^−1^) and v _1_PO_4_ bands. High values indicate higher levels of calcium phosphate (CaHPO_4_, CaHPO_4_ ・ _2_H_2_O).
- Collagen structural integrity = intensity ratio of 1640 and 1670 cm^−1^ in amide I. High values indicate more robust collagen structure.

### Analysis of serum for assessing oxidant and anti-oxidant stress

The reactive oxygen metabolites (d-ROMs) test (Diacron International, Grosseto, Italy) was performed on serum from all rats (n = 12), as reported in detail elsewhere^22^. The d-ROM test was based on levels of serum reactive oxygen metabolites measured using a spectrophotometer. The Carratelli unit (CARR U) was used as the measurement unit.

In addition, the OXY-adsorbent test (Diacron International, Grosseto, Italy) was performed on serum from all rats as reported in detail elsewhere^22^. The OXY-adsorbent test is based on total serum anti-oxidant capacity, measured using a spectrophotometer. This test evaluates the serum capacity required to oppose the massive oxidative action of a hypochlorous acid (HClO) solution. Total anti-oxidant capacity was expressed as μmol of HClO consumed by 1 mL of sample (μmol HClO/mL).

Furthermore, the oxidative-INDEX was calculated from the results of the d-ROM and OXY-adsorbent tests to indicate the balance between serum reactive oxygen metabolites and anti-oxidants. To incorporate parameters with different measurement units, the standardized values of the ROM and OXY-adsorbent tests were determined using the formula developed by Vassale *et al*.^22^:

$$sv \mbox{-} var=(v \mbox{-} var-m \mbox{-} var)/sd \mbox{-} var$$

where *sv-var* is the standardized value of a given parameter, *v-var* is its original value, and *m-var* and *sd-var* are the mean and standard deviation of the parameter in both groups (n = 12).

The standardized oxidative-INDEX value was calculated by subtracting the *sv-var* of the OXY-adsorbent test result from the *sv-var* of the ROM test result. High oxidative-INDEX values indicate high oxidative stress in the blood.

### Statistical analysis

Statistical analysis was performed using JMP^®^ (version 13.3.0, SAS institute Inc. Cray, NC, USA). The statistical data are provided as mean and standard deviation. The t-test was used to compare data from experiments using multiple treatments. A p-value less than 0.05 indicated a significant difference.

## Results

### MicroCT analysis and pQCT for assessing osteoporotic development

**Figure 2:**
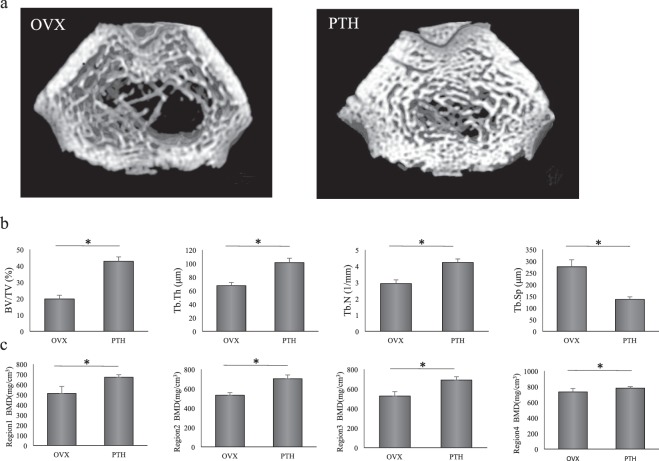
Assessment of PTH in the treatment of osteoporosis in the right femur. (**a**) Coronal view of three-dimensional images of the femur obtained using microCT. The femurs of OVX and PTH rats are shown. (**b**) Parameters of cancellous bone obtained using microCT. Measurements were performed on an area 1.5 to 3.5 mm proximal to the growth plate at one end of the distal metaphysis of the femur. The target region was examined for bone volume (BV/TV), and the thickness (Tb.Th), number (Tb.N), and separation (Tb.Sp) of the trabeculae. *P < 0.05. (**c**) Bone mineral density (BMD) of the femur obtained using pQCT. These values were measured at regions 1, 2, 3, and 12 mm proximal to the growth plate. *P < 0.05.

MicroCT images of femurs from the groups were used to assess various bone morphometric indicators (Fig. 2a). Trabecular data (3D microCT) showed that BV/TV and Tb. Th were significantly increased in PTH rats compared to OVX rats, while Tb. N an d Tb. Sp were similar between the two groups (Fig. 2b).

To evaluate the effect of PTH on bone calcification, BMD at different regions in the femur was investigated using pQCT. BMD was significantly increased in PTH rats compared to OVX rats at regions 1 to 4 (Fig. 2c).

### MicroCT analysis to assess the histomorphometry of new mandibular bone

**Figure 3:**
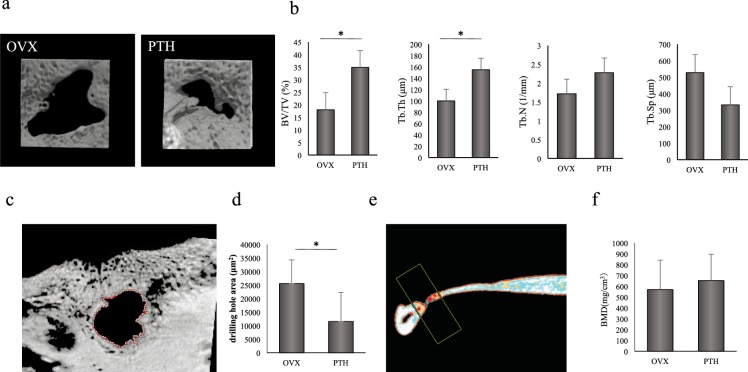
Analysis of BMD and structural changes in newly formed bone of the mandible. (**a**) Three-dimensional images of the mandible obtained using microCT. The mandibles of OVX and PTH rats are shown. (**b**) Parameters of cancellous bone obtained using microCT. Measurement was performed on an area 1.5 to 3.5 mm proximal to the growth plate located at one end of the distal metaphysis of the femur. The target region was examined for bone volume (BV/TV), and the thickness (Tb.Th), number (Tb.N), and separation (Tb.Sp) of trabeculae. *P < 0.05. (**c**) Image of the hole in the mandible extracted from microCT images. An image in which the area of the drilled hole was minimized was extracted from microCT images and examined using bone analysis software. (**d**) Area of the hole (μm2) in the mandible obtained using microCT. The area of the hole in PTH rats was significantly decreased compared to that in OVX rats. *P < 0.05. (**e**) Measured area of the mandible obtained using pQCT. Image obtained using pQCT showing the measured area of the removed mandible 21 days after drilling. Measurement was performed on a rectangular area with long edges at a distance of 1.3 mm from the center of the drilled hole. (**f**) BMD of the newly formed bone in the mandible obtained using pQCT. The BMD of newly formed bone was not significantly different between the groups.

MicroCT images of the mandibles from all groups were evaluated to assess various bone morphometric indicators (Fig. 3a). 3D microCT data showed that BV/TV and Tb. Th were significantly increased in PTH rats compared to OVX rats, while Tb. N and Tb. Sp were similar between the two groups (Fig. 3b). We also measured the area of the drilled hole (μm^2^) by extracting an image from the microCT images in which the area of the drilled hole was minimized (Fig. 3c). This analysis showed that the area of the drilled hole was significantly smaller in PTH than OVX rats (Fig. 3d).

### pQCT analysis of newly formed mandibular bone

To determine the effect of PTH on calcification of new bone in the mandible, the BMD in newly formed mandibular bone (Fig. 3e) was measured using pQCT after treatment. The BMD of newly formed bone did not significantly differ between groups (Fig. 3f).

### Raman microspectroscopic analysis of newly formed bone of the mandible

**Figure 4:**
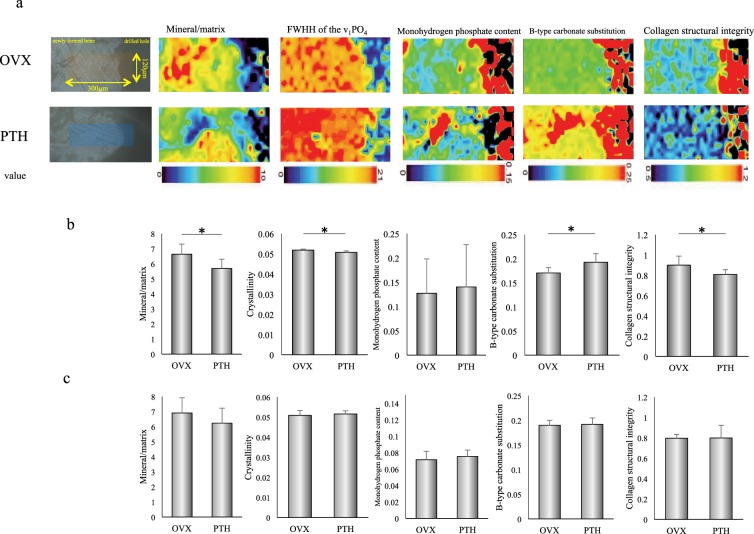
Bone quality parameters obtained from Raman spectra. (**a**) Transmission electron microscopy images showing the site used for the mapped image and the mapped images of bone quality parameters. The site used for mapping, with spectra obtained at intervals of 10 µm within the 120 µm × 300 µm area, including the drilled hole. The mapped images of the mandible were colored based on the value of bone quality parameters from the drilled hole to the newly formed bone in OVX and PTH rats. Measured bone quality parameters were mineral/matrix ratio, full-width at half-height (FWHH) of the v_1_PO_4_ band (indicator of crystallinity), monohydrogen phosphate content, B-type carbonate substitution, and collagen structural integrity. In these mapped images, blue indicates a lower value and red indicates a higher value. Compared to OVX rats, PTH rats showed a decrease in the mineral/matrix ratio and collagen structural integrity, and an increase in the FWHH of the v_1_PO_4_ band and B-type carbonate substitution. (**b**,**c**) Bone parameters obtained from Raman spectra. Similar to those for the mapped images, measured parameters at 90 μm (**b**) and 2 mm (**c**) from the bone edge were the mineral/matrix ratio, crystallinity, B-type carbonate substitution, monohydrogen phosphate content, and collagen structural integrity in OVX and PTH rats. *P < 0.05.

The mapped images of the mandibles from each group obtained using Raman microspectroscopy were employed to assess various indicators of bone quality (Fig. 4a). A mapped image was taken of the sample area, which included the area of bone loss and newly formed bone, and colored according to bone quality parameter values within the sample area (width × length: 300 μm × 120 μm) using a scanning step size of 10 μm (width × length: 30 × 12 points) (Fig. 4a). This analysis indicated that newly formed mandibular bone in PTH rats had reduced mineralization and a deterioration in mineral and collagen quality compared to OVX rats.

Subsequently, to assess bone quality parameters, measurements were performed at four points 90 μm and 2 mm external to the inner circumference of the hole in the mandible. At 90 μm external to the inner circumference of the hole, the mineral/matrix ratio, crystallinity and collagen structural characteristics were significantly decreased, and B-type carbonate substitution was significantly increased in PTH compared to OVX rats. No significant difference in monohydrogen phosphate content was observed in OVX and PTH rats (Fig. 4b).

At 2 mm external to the inner circumference of the drilled hole, no significant difference was observed in the mineral/matrix ratio, crystallinity, monohydrogen phosphate content, B-type carbonate substitution or collagen structural integrity (Fig. 4c).

### Analysis of oxidation in blood

**Figure 5:**
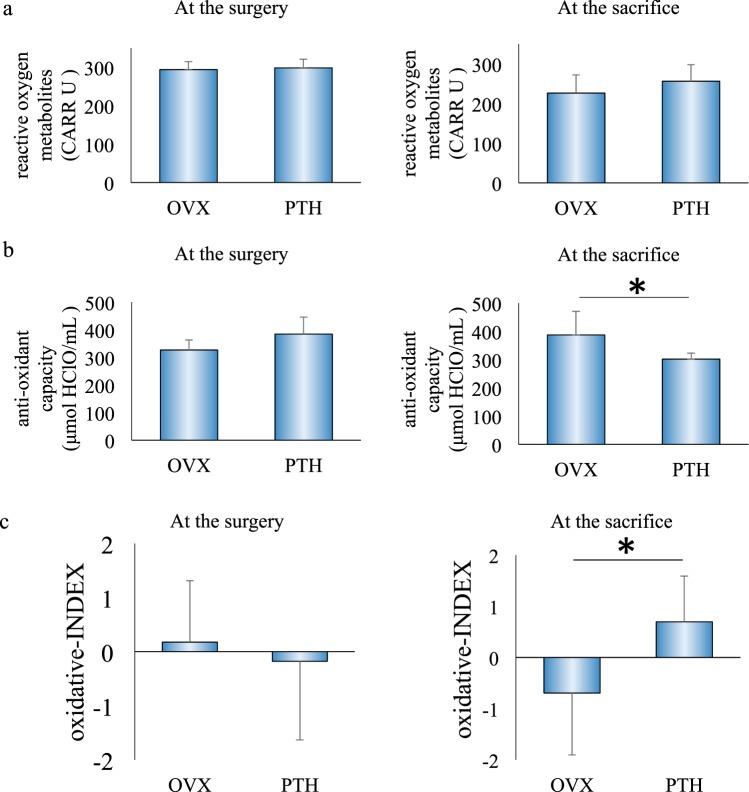
Analysis of serum for assessing oxidant and anti-oxidant stress. At the time of surgery, levels of serum reactive oxygen metabolites, total anti-oxidant capacity and the oxidative-INDEX did not significantly differ between the groups. At sacrifice, while levels of serum reactive oxygen metabolites did not significantly differ between the groups, serum total anti-oxidant capacity was significantly decreased, and the oxidative-INDEX was increased in PTH rats compared to OVX rats. *P < 0.05.

Serum was collected from rats to examine the degree of oxidation at the time of surgery and sacrifice. At the time of surgery, levels of serum reactive oxygen metabolites, total anti-oxidant capacity and the oxidative-INDEX did not significantly differ between the groups. At sacrifice, while the levels of serum reactive oxygen metabolites did not significantly differ, total anti-oxidant capacity in serum was significantly decreased in PTH rats compared to OVX rats and oxidative-INDEX was increased (Fig. 5).

## Discussion

While several reports have suggested that PTH improves the bone density and structural characteristics and enhances the healing of newly formed jawbone^6–9^, these studies did not examine the effects of PTH on the material characteristics of the newly formed jawbone. Deterioration of material characteristics is known to occur through the activation of osteoclasts and increased levels of AGEs due to oxidative stress^3,4^. We demonstrated that PTH improved the bone mass and structural characteristics of newly formed mandibular bone. However, PTH administration reduced mineralization and crystallinity while increasing carbonates, and decreased the collagen structural integrity of bone matrix of newly formed mandibular bone. Moreover, PTH produced an increase in the oxidative-INDEX accompanied by a decrease in anti-oxidative ability. Our findings therefore suggest that PTH administration caused a deterioration in the mineral quality and collagen quality of newly formed mandibular bone due to oxidation.

Oxidation increases the levels of AGEs, which decrease bone strength and toughness, and induce osteoclastic activity^3,4^. We found that rats administered PTH showed a decrease in anti-oxidative ability and an increase in the oxidative-INDEX. The OXY-adsorbent test measures anti-oxidative ability, which is an indicator of the presence of anti-oxidants in the serum. In particular, melatonin, which is secreted from the pineal gland into the blood, is known to be a powerful anti-oxidant^23^. Previous studies have shown an interaction between PTH and melatonin^24,25^. Specifically, serum melatonin levels changed in inverse proportion to serum PTH levels^24^. Our present results may suggest that daily administration of PTH inhibits the secretion of anti-oxidants like melatonin, which may in turn reduce anti-oxidative capacity and increase the oxidative-INDEX. Indeed, serum analysis revealed that PTH promoted oxidation while lowering anti-oxidative ability.

Bone is a two-phase composite comprising a mineral phase, which provides stiffness by resisting transformation, and collagen matrix, which provides toughness and ductility (contractile characteristic) by resisting pressure and tension^26^. The mineral/matrix ratio measured using Raman microspectroscopy can be employed as an index of calcification. In rodents, the mineral/matrix ratio correlates well with bending stiffness and breakage point of the upper arm, independently of BMD^27^. In the present study, PTH administration significantly decreased the mineral/matrix ratio compared to vehicle administration, suggesting that PTH suppresses mineralization in newly formed mandibular bone.

Bone mineral quality is measured by crystallinity, the maturity of crystals and the degree of replacement of the crystal structure^28^. In Raman spectroscopy, mineral quality can be evaluated using three bone quality parameters, namely crystallinity, B-type carbonate substitution and monohydrogen phosphate content. Crystallinity, as measured using Raman microspectroscopy, is a reflection of both lattice order (completeness of the apatite crystal domain) as well as the relative size/distortion of crystals^28^. B-type carbonate substitution assesses the substitution of carbonate ions (CO_3_^−^) for phosphoric acid (PO_4_) in the crystal structure. CO_3_^−^ has greater solubility than PO_4_ with regard to apatite crystal structure, and is an unstable fraction of bone^28,29^. Increased B-type carbonate substitution is known to be correlated with the distortion of lattice structure, and widens the PO_4_ band in the Raman spectrum, decreasing crystallinity parameter values^28^. On this basis, the significant increase in B-type carbonate and decrease in crystallinity in PTH rats on comparison with OVX rats observed in the present study might therefore indicate a distortion of the lattice structure of newly formed bone. In addition, monohydrogen phosphate content can be used to measure levels of calcium phosphate (CaHPO4, CaHPO4 · 2H2O), which is a precursor to the crystal structure of hydroxyapatite. Labile nonaptic domains (HPO_4_, PO_4_, CO_3_), which are related to a reduction in bone strength, decrease during bone tissue maturation^29^, indicating that they are correlated with mineral maturity. In the present study, while there was no difference in the amount of calcium phosphate, there was an increase in B-type carbonate substitution in PTH rats, suggesting that the decrease in mineral maturity might be induced by an increase in B-type carbonate substitution. These results suggest that ovariectomy and PTH influence the mineral quality of newly formed mandibular bone during bone healing.

Bone collagen plays a key role in the toughness of bone^26^. Raman microspectroscopy is one method used to evaluate collagen quality. A peak ratio of 1640/1670 has been previously shown to be suitable for the assessment of collagen quality and is reflective of changes in the collagen secondary structure such as collagen crosslinks; or more specifically, the transition from an ordered structure to one less ordered^20^. Oxidative stress is known to reduce the collagen structural integrity by enhancing the formation of non-enzymatic collagen crosslinks, which are a type of AGE^3^. In the present study, an increase in the oxidative-INDEX was observed after PTH administration, indicating a decrease in the completeness of the collagen structure. In addition, Raman microspectroscopy demonstrated a deterioration in the collagen structural integrity of PTH rats compared to OVX rats. These results may suggest that the production of AGEs following oxidative stress causes a deterioration in collagen crosslinks in newly formed bone.

In this experiment, assessment of newly formed bone was done using a 2.0 mm hole. A previous study reported that a critical defect size for the mandible of Wistar rats was 4.0 mm^30^. However, producing a 4.0 mm defect may result in damage to the inferior alveolar nerve vascular bundle, which controls sensory innervation of the lower lip and mandibular gums. Because this would likely have a substantial impact on the results, we made smaller 2.0 mm holes for the cortical size defect. In addition, we confirmed that the holes were not blocked and the inferior alveolar nerve vasculature was not injured in all specimens using microCT at the time of sacrifice.

In dental implant treatment, the newly formed bone is interposed between the drilled hole and the implant fixture to achieve osteointegration. That is, the performance of dental implant treatment is directly linked to the bone quality of the newly formed bone. In particular, the bone quality of newly formed bone at early stages after dental implant surgery is very important in the case of immediate implant placement, in which the implant body is occluded immediately after tooth extraction and dental implant replacement. Previous studies have demonstrated that the quality of the mineral and matrix plays a critical role in the stable osteointegration of dental implants^31,32^. As PTH is expected to improve the bone density and microstructure of the bone around the dental implant, researchers are attempting to adapt it for dental implant treatment^33,34^. However, the quality of the newly formed bone mineral and matrix following PTH treatment remains unclear. To our knowledge, the only report that has demonstrated the effect of PTH on the material characteristics of newly formed bone examined bone quality parameters in a murine tibial fracture model using Raman spectroscopy^10^. The researchers found that administration of 25 μg/kg PTH for two weeks from 8 days after fracture operation decreased B-type carbonate substitution but had no effect on matrix quality or collagen orientation^10^. In contrast, we found that PTH improved the bone mass and microstructure of newly formed bone, but caused deterioration in the quality of the mineral and matrix. These differences might be explained by differences in the characteristics of jaw and tibial bone, the duration of administration, the dose of PTH administered and the ossification types. Unique characteristics of the jawbone include the high loading it encounters and faster bone turnover^35^. Our present results demonstrate that PTH may have a unique effect on newly formed jawbone.

Furthermore, although PTH improves bone mass and structural characteristics, which are advantageous for the osteointegration of a dental implant, it causes a deterioration in the quality of the mineral and matrix, thereby decreasing bone strength. This is disadvantageous for osteointegration of a dental implant. Therefore, further studies are required to adapt PTH to dental implant treatment.

In addition, the mechanism of this adverse impact on mineral and matrix is unclear. We speculate the following. It is known that PTH, which promotes fast bone turnover, promotes an increase in the mass of newly formed bone^6–9^. Mandibular bone is characterized by its faster bone turnover than other skeletal bone^35^. Together, this characteristic and the effect of PTH results in excessive bone turnover in the mandibular bone. In addition, this study showed that PTH causes a decrease in serum oxidation by decreasing antioxidant capacity. Oxidation causes deterioration in collagen structures and activates osteoclasts^3^. The combined effect of these factors, including the unique characteristics of mandibular bone and oxidation, may result in increased bone mass and improved structural characteristics, but also reduced mineralization and deteriorated collagen in newly formed mandibular bone.

In conclusion, while intermittent administration of PTH promotes the healing of newly formed mandibular bone by improving the microstructure and increasing bone mass, it also promotes oxidation, which depreciates the quality of bone minerals and bone collagen. While the outcome of mandibular dental implant treatment depends on bone mass and bone quality, osteoporosis worsens bone mass and bone quality in the jaw. It is therefore necessary to determine how PTH affects bone quality, which will in turn influence the treatment outcomes of jawbone surgeries, including dental implant surgeries, in the future. If changes in bone quality following PTH administration can improve the outcome of dental implant treatments, it may be possible to use PTH during dental implant treatment for osteoporosis patients.
